# OVERVIEW OF COVID-19 STUDY WITH OLDER ADULTS IN PUERTO RICO

**DOI:** 10.1093/geroni/igac059.619

**Published:** 2022-12-20

**Authors:** Thomas Buckley, Denise Burnette

**Affiliations:** University of Pittsburgh, Pittsburgh, Pennsylvania, United States; Virginia Commonwealth University, Richmond, Virginia, United States

## Abstract

This presentation will provide an overview of our study with older adults in Puerto Rico during COVID-19. From January through December, 2021, we conducted telephone and face-to-face interviews with a nonprobability sample of 213 adults aged 60+ in 21 of the island’s 78 municipalities. Average age of participants was 71.9 (SD=8.8), 55.9% were female and 55.4% had annual household incomes under $12,500. About two in five older adults reported feeling lonely (39.4%) and poor/fair health (39.9%). Fully 65.3% were overweight or obese, 59% reported at least one chronic disease (59.4%) and 25.8% had delayed medical care during COVID-19. KAP index scores were reasonably high and 97.2% of participants were either vaccinated or planned to be. We will also discuss the development, translation and adaptation of the data collection instrument and the important social, economic and political backdrops of successful public health measures during the pandemic in Puerto Rico.

